# Development of Scientific Fishery Biomass Estimator: System Design and Prototyping

**DOI:** 10.3390/s20216095

**Published:** 2020-10-27

**Authors:** Pranesh Sthapit, MinSeok Kim, Donhyug Kang, Kiseon Kim

**Affiliations:** 1 Gwangju Institute of Science and Technology, Gwangju 61005, Korea; moodyblue1014@gmail.com; 2 Korea Institute of Ocean Science and Technology, Busan 49111, Korea; dhkang@kiost.ac.kr

**Keywords:** biomass, closed pen, fish detection, fish counter, FPGA, hydroacoustic, echosounder, prototyping

## Abstract

This paper presents a new compact single beam advanced echosounder system designed to estimate fish count in real time. The proposed device is a standalone system, which consists of a transducer, a processing unit, a keypad, and a display unit to show output. A fish counting algorithm was developed and implemented in the device. The device is capable of performing all the functions required for fish abundance estimation including target strength calculation, simultaneous echo integration, and echogram generation. During operation, the device analyzes ping data continuously and calculates various parameters in real time while simultaneously displaying the echogram and results on the screen. The device has been evaluated by technical verification in a lab and on-site experiments. The experimental results demonstrate that the proposed device is on par with a commercial echosounder and is capable of accurately estimating the fish abundance. The proposed device is beneficial for fish management.

## 1. Introduction

Fish abundance estimation is an open problem but a necessity in fish farming. A reliable estimation of fish under cultivation aids with delivering the optimum care for fish, thus ensuing the good health as well as proper growth of the species [1,2,3,4]. Furthermore, the knowledge of fish amount helps in the proper management of inventory related to fish such as fish food and prevents under or overfeeding [3,4]. Another area where knowledge of the fish count would be beneficial is for the insurance companies, as they do not have any other option than believing in farmers’ words. Generally, the approximate fish count is known at the initial phase of farming. However, over time, the amount of fish decreases significantly. However, the early count or rough approximation is generally used for fish management as there is no reliable way of tracking them. Therefore, a portable device for estimating fish count in real time is proposed in this paper.

Over the past few decades, various algorithms and techniques have been proposed and used to estimate fish. To illustrate, acoustics echo-sounding, machine vision-based technique, environmental DNA based method, and resistivity counting technique are some well-known techniques [5]. However, because of its accuracy and noninvasive nature, acoustic echo-sounding is a popular choice for fish count estimation [6]. In a typical echosounder, the transmitter emits a ping (acoustic signal) and the received echo signals from targets are analyzed. The fundamental principle is the detection of reflected energy from a target. It is not uncommon to use sonars and fish finders to locate fish in the commercial fishing operations [6]. The academic as well as commercial use of acoustics for estimating fish abundance have been widespread [7,8,9,10,11,12,13,14,15,16,17,18,19]. Although fish have been the main focus, squids and other marine organisms have also been investigated. Modern echosounder implements the echo integration to analyze fish and their behavior [10,11,12,13,14,15]. Even though the echo integration method has been extensively employed in open ocean, however, when the method is employed in the farming cages, it offers new challenges [16,17,18,19,20]. Firstly, the fish are closer to transducer, therefore, classical Time Varying Gain (TVG) correction could be inappropriate [16]. Secondly, the reverberation of the acoustical signal from the boundaries of cage need to be considered [17,21,22]. Another problem that needs to be addressed is removing the cage signal from the received echo [20]. Similarly, the stability of the transducer is another issue as the signal quality highly depends on its orientation [17,22].

The main goal of this research is to build a cost-effective, portable, compact, and stand-alone scientific echosounder (SE) that can accurately estimate fish in a closed pen. Unlike the most state-of-the-art fish counting method, the proposed device does not need separate hardware and software for fish counting. Everything required for the fish counting is built on a single device. This paper details our novel device, its design process, the hardware and software components used, experiments performed, and the test results. Our compact design consists of a keypad, a display, FPGA, and a processing unit. Even though few ready-made commercial components are used, most of the hardware, software, and algorithms are developed and implemented in our lab. The device is built using the fundamental principles of the standard echosounder, but with enhancement. The proposed device implements programmable gain to improve dynamic range, time-variable gain to compensate transmission loss, high-speed ADC to achieve high depth resolution, bottom detection algorithm to detect net bottom and sea bed, and a novel fish counting algorithm to count fish. To our best knowledge, this is the first portable echosounder that has a built-in fish counting mechanism. The prototype device was evaluated and compared with a commercial echosounder. Our experimental results show that the performance of the device is on par with the commercial echosounder. Furthermore, our on-site experimental results show that the device could estimate the fish amount accurately. We note that even though the device has fish counting ability, it can still be used as a normal echosounder. The rest of the paper describes the design and implementation of the proposed device in detail.

## 2. Theoretical Aspects

### 2.1. Echo-Sounder

The modern echosounder device needs a personal computer (PC) to analyze and visualize data; therefore, it comes with the software. The installed software in the PC can display the received signal graphically as an “echogram”. The echosounder mainly consists of four components: transducer, transmitter, receiver, and display unit. The transmitter generates an electrical pulse that is transformed to sound waves by the transducer and propagate through the water. The reflected signal from the targets in water are sampled by the transducer again, changed back to electrical signal, and echograms are generated. SE for acoustic assessment operates on the same fundamental principles, but with the precision required for scientific study [6,23]. The fundamental principles of target detection in water with an echosounder are illustrated by the sonar equation as [6]

(1) EV=SL+G−40LogR−2aR+TS+2B(θ,ϕ)

where echo voltage (EV) is the measured echo voltage in dB, transmitted source level (SL) is in dB re

1 μPa at 1 m, receive gain (*G*) is in dB/μPa at 1 m, *R* is range in meters, 40logR is the two-way spreading loss in dB, sound attenuation coefficient (*a*) is in dB/m, acoustic target strength (TS) is in dB, and B(θ,ϕ) is the transducer directivity pattern function. In the above equation, if EV is larger than noise, the target can be detected. The target detection is affected by the noise produced from various sources such as a ship, electronic equipment, or sea. The ability to detect a target can be improved with less propagation loss, larger source level, and location of the target to the center of the transducer beam. The sound wave gets attenuated as it propagates. The propagation loss arises from the spreading of the beam as it propagates, and through sound absorption. Generally, sound absorption is higher with increasing salinity and sound frequencies. Generally, SE automatically corrects for the propagation loss by employing “time-varied-gain”.

### 2.2. Target Strength, Volume and Area Backscattering Coefficient, and Fish Density Estimation

In the fish abundance estimation, TS is used as a scaling factor [24]. TS is scattering properties of an organism (in dB). $TS=10log(\sigma_{bs})$, where $\sigma_{bs}$ is acoustic backscattering cross-section reflected by a target. The echo reflected from individual targets are blended to form an integrated received signal when there are many targets. The volume backscattering coefficient, $s_{v}$, is calculated as $s_{v}=\frac{\sum \sigma_{bs}}{V}$, where the sampled volume is denoted by *V*. The area backscattering coefficient, $s_{a}$, is a measurement of the reflected energy from a layer between two depths in the water column. It is the integration of $s_{v}$ w.r.t depth through the layer r1 and r2 and is given by

(2) $$s_{a}=\int_{r1}^{r2}s_{v}dr$$

If we know the total energy reflected by all fish and the energy reflected by a single fish, their ratio gives an estimation of the fish count. Assuming that all fish are of the same size, the area density ($\rho_{a}$) can be calculated as $\rho_{a}=\frac{s_{a}}{\sigma_{bs}}$. However, the accuracy is dependent on the estimation of $s_{a}$ and $\sigma_{bs}$. Furthermore, since multiple targets of various sizes and spices can exist in the water column, <$\sigma_{bs}$> should be calculated and used.

## 3. Hardware

### 3.1. Overview of the System

We use the term Scientific Fishery Biomass Estimator (SFBE) to refer to the proposed device. The first and foremost design goal of SFBE is that it should be a complete stand-alone fish counting system. This means that it should perform fish counting by itself without needing any hardware or software. The other major design requirements of the SFBE are as follows: (i) it should be cost-effective, reliable, portable, and lightweight; (ii) it should have a user-friendly interface; (iii) it should collect and display important fish information; (iv) it should record echogram; (v) it should detect target up to 100 m of depth; and (vi) it should support data communication with a computer.

**Figure 1:**
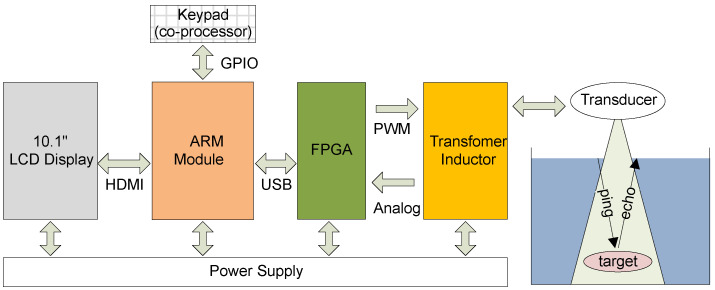
Architecture of the SFBE device.

**Figure 2:**
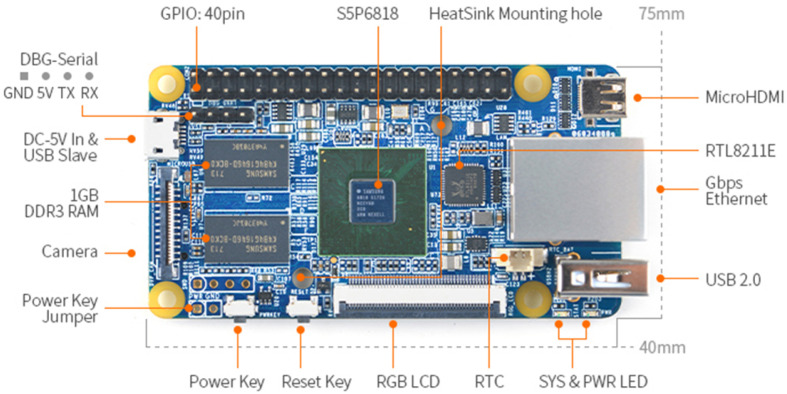
ARM module.

The architecture of the proposed device is shown in Figure 1. The SFBE consists mainly of a keypad, FPGA, processing unit, transducer, and a display. The SFBE displays a graphical user interface (GUI) on the screen to interact with users. The inputs to SFBE are given via the keypad. The processing unit handles acoustic signal processing and computations. The FPGA handles the analog I/O signals to and from the device. The power supply unit supplies the required power to each part. The power supply for different units is isolated to eliminate noise. The ARM module [25] shown in Figure 2 is the main processing unit and is responsible for controlling overall hardware and software. The specification of the module is illustrated in Table 1. The ARM module runs on the optimized lightweight Linux operating system. The ARM module is responsible for the following main functions: (i) FPGA control and ADC sampling; (ii) signal processing and computations; and (iii) GUI related functions.

**Figure 3:**
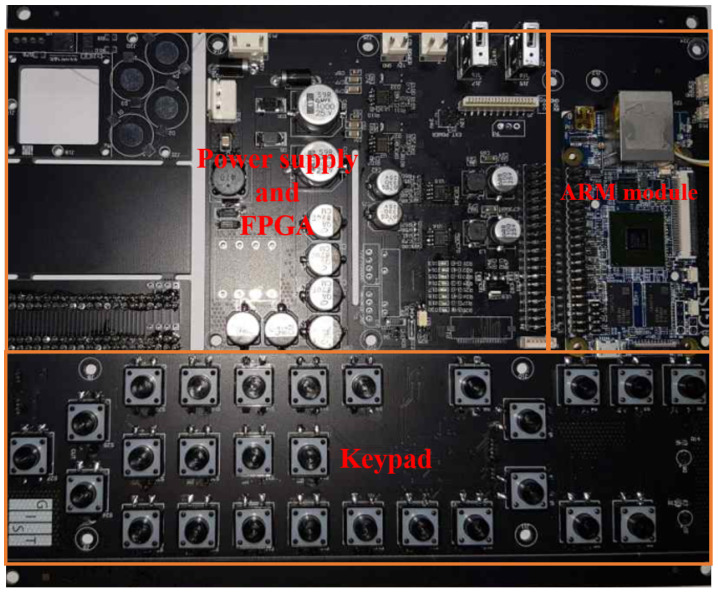
The printed circuit board of the motherboard used in SFBE.

Table 2 illustrates the pin description of the ARM module. Only the pins shown in red are used. Power is supplied through pins 2, 4, 6, and 25. The power supply of 5 V, 2 A is supplied to the module for the stable operation. Figure 3 illustrates the designed motherboard of SFBE, which inter-connects all the hardware components. The motherboard is built on a double-sided printed circuit board. The top middle part of the motherboard housed the power supply and the FGPA is placed on the other side. The motherboard connects the keypad (bottom half) and FPGA with the ARM module (middle right part). The ARM module and the FPGA are connected with the serial peripheral interface (SPI) via pins 19, 21, 23, and 24. The ADC data are transmitted at the maximum speed of 250 Mbps whereas regular data at 200 Mbps. The ARM module uses the general-purpose input/output (GPIO) for handshaking with the FPGA as follows. Setting pin no. 29 to HIGH operates the FPGA in the configuration mode to set variables such as pulse interval and pulse length. Similarly, resetting pin 29 to LOW switches the FPGA into the transmission mode during which ADC samples are transmitted to the ARM module in burst mode. Pin No. 31 is used as a trigger signal to generate an acoustic pulse and ADC sampling and setting it to HIGH starts pulse generation. Similarly, resetting it to LOW stops all FPGA operations and switches FPGA to the standby mode.

The LCD display (1920 × 1088 pixels) is connected to the ARM module via HDMI 1.4 A standard. The keypad is designed and implemented separately using Stm32F103CB 32-bit 72 MHz [26] co-processor in order to reduce the load of the ARM module. The co-processor detects the key-press and notifies the ARM module through a universal asynchronous receiver transmitter (UART) using pin Nos. 8 and 10.

Along with all the functionality of a typical echosounder, the proposed system adds two extra features. SFBE supports the temperature sensor interface using the 1-wire protocol and the camera interface using USB video class (UVC) protocol. The temperature sensor interface is employed to automatically record water temperature, which is necessary to calculate the sound speed. Similarly, the camera feature enables easy visualization of the underwater environment on the screen.

### 3.2. Power Supply

**Figure 4:**
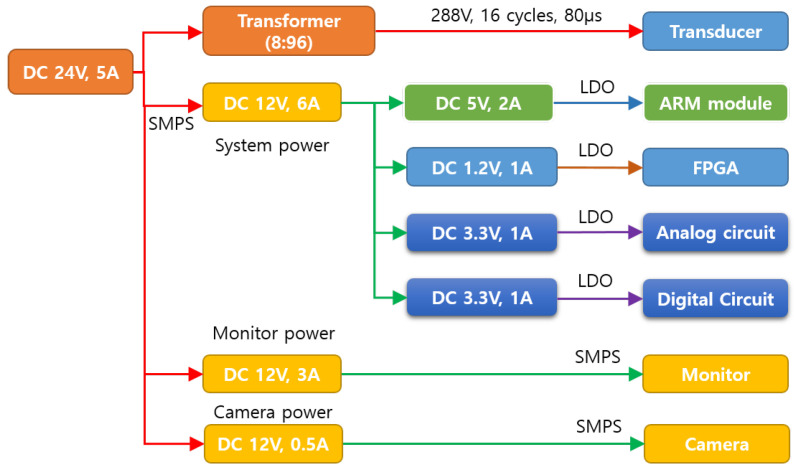
The block diagram of power supply of SFBE.

The power supply of SFBE is shown in Figure 4. The power supply is designed with an input power of DC 24 V and 5 A. The input supply to each module is designed and developed separately to minimize the noise among components. The main input DC power of 24 V is used to ensure the generation of acoustic signals with enough power. Accordingly, 12 V DC was generated by using the step-down switched-mode power supply (SMPS) technique. However, low-dropout (LDO) supplies are given to the ARM module, FPGA, analog circuits, and digital circuits.

### 3.3. FPGA

**Figure 5:**
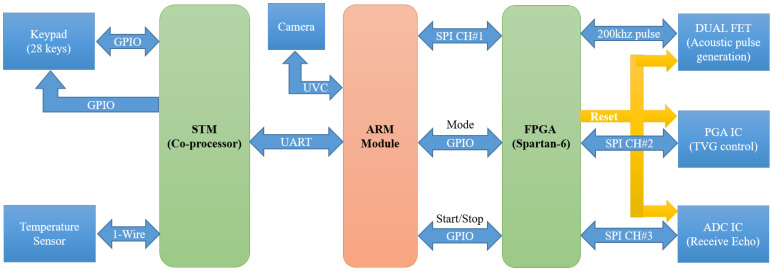
Block diagram of handshaking and communication used by the ARM module with the FPGA and co-processor.

FPGA module is developed using Xilinx’s Spartan-6 FPGA (XC6SLX9-2TQG144C) [27]. The FPGA is responsible for the 200 kHz acoustic wave generation and ADC sampling of received echos. The main function of FPGA is the synchronization of the input and the output acoustic signals. By using handshaking signals from the ARM module, it generates acoustic pulses as well as samples the received echo signals. The sampled ADC data are transmitted to the ARM module on demand at high speed via the SPI bus. Figure 5 shows the architecture of handshaking and communication used by FPGA. As shown in the figure, FPGA uses three SPI channels for communication. SPI channel 1 is used to communicate with the ARM module regarding TVG control, pulse length control, and ADC transmission and reception, and FPGA operates in SPI Slave Mode3. SPI channel 2 is used to communicate with PGA IC for Coarse TVG control purposes, during which FPGA operates in SPI master Mode3. SPI channel 3 is used for controlling ADC IC and during which FPGA operates in SPI master Mode3.

### 3.4. Analog Circuit

**Figure 6:**
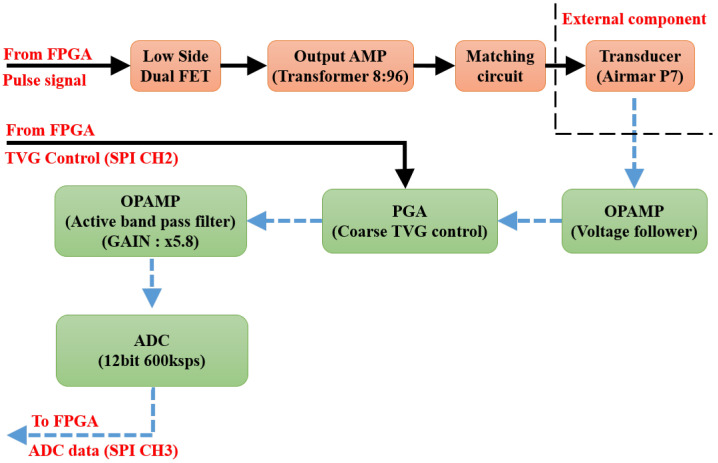
Analog circuit block diagram of SFBE.

**Figure 7:**
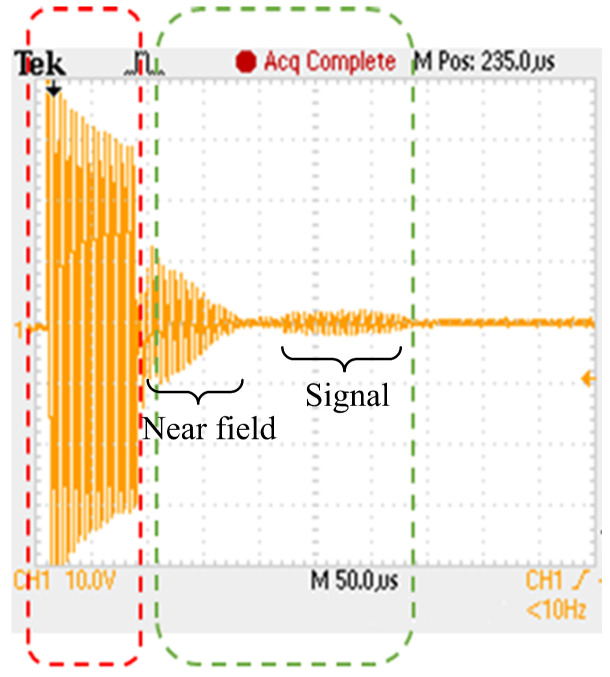
The ping and echo signals from SFBE recorded by an oscilloscope.

The block diagram of the analog circuit used in SFBE is shown in Figure 6. The key function of the analog circuit is the generation and reception of the acoustic signal. The function of each block in the diagram is as follows. The Low Side Dual FET block generates a square wave of 200 kHz using the pulse from the FPGA. Two field-effect transistors (FET) are used to generate ±24 V. The Output AMP block is responsible for amplifying the input signal 12 times with a winding ratio of 8:96. The output voltage is 288 V AC. The Matching circuit block is an L, C series circuit used for the impedance matching of the transducer. The commercial Airmar P7 [28] transducer is used in our prototype device to send and receive acoustic signals. The transducer is operated at 200 kHz and has a beamwidth of 14°. The OPAMP (voltage follower) block amplifies the input signal, which prevents the echo signal being attenuated by the filter circuit. Furthermore, the received signal is further amplified using a Programmable Gain Amplifier (PGA) to maximize the output dynamic range of the receiver. FPGA controls PGA using SPI Channel 2 and TVG is applied depending on the time of the received signal. The TVG feature in SFBE compensate for transmission loss and make the echo level independent of a target range. The OPAMP (Active Band Pass Filter Amplifier) blocks filters out noise. Active filters are applied using OPAMP, and only the signal from 175 to 225 kHz is band passed and amplified by 5.8 times. The analog to digital converter (12-bit ADC, LTC2365) converts the analog input echo signal into digital data. Only the half-wave of the received acoustic signal of 200 kHz is over-sampled at 600 kHz, whereas the other half-wave is ignored. The data sampled at 600 kHz is down sampled to 200 kHz by software after peak detection. Oversampling is performed to improve resolution and signal-to-noise ratio [29]. Since every reflected wave is sampled, our device gives the depth resolution of 3.75 mm [30]. Figure 7 illustrates the transmitted ping and the received echo recorded by an oscilloscope.

### 3.5. Co-Processor

The co-processor module, built using Stm32F103CB, continuously monitors the keypad and notifies a keypress to the ARM module. In addition, the co-processor monitors the system for the possible failure and monitors the operation status of the ARM processor. The functions of co-process are (i) scan the user keypad; (ii) data collection from temperature sensor using 1-wire communication protocol; (iii) main power supply monitoring, (iv) system recovery by monitoring the ARM module and rebooting on failure; and (v) exchange data with ARM module using the UART communication.

## 4. Software

**Figure 8:**
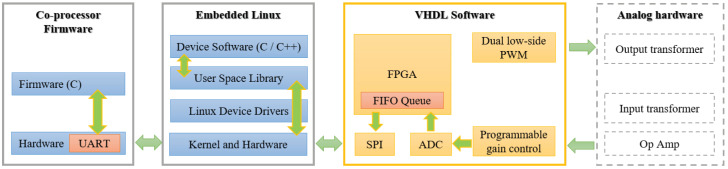
The block diagram of software components used in SFBE.

The block diagram of SFBE software components is shown in Figure 8. The software consists of mainly three components: co-processor firmware, the device software, and FPGA VHDL (Field Programmable Gate Arrays Very High Speed Integrated Circuit Hardware Description Language) software. The device software is developed using C/C++ and runs under a Linux environment. It is the main software responsible for the overall function of the system. The co-processor firmware is responsible for the keyboard scanning, the temperature sensor communication, and system monitoring. The firmware runs in ARM-MCU and is written in C language. FPGA VHDL software is responsible for input/output of the analog acoustic signal.

### 4.1. FPGA VHDL Software

**Figure 9:**
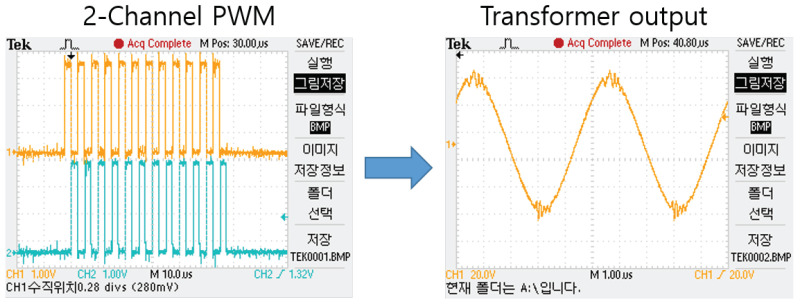
200 kHz acoustic signal generation in SFBE.

The VHDL is responsible for the following functions: (i) Analog data synchronization and management; (ii) Generation of 200 kHz acoustic wave; (iii) TVG control; (iv) ADC sampling; (v) ADC data FIFO I/O buffer management; and (vi) Send/receive ADC data to/from ARM module. To execute the fore-mentioned functions, FPGA uses various handshaking signals and I/O ports. In addition, FPGA generates various output clocks for different purposes as illustrated in Table 3. The acoustic signal is generated by using dual N-channel FET with PWM of 50% duty cycle on two channels which is then fed to the transformer to generate a 200 kHz acoustic wave. The overall process of wave generation is illustrated in Figure 9. To prevent from the zero-crossing collision, reduce heat generation, and to improve the performance of the transformer, a dead-zone delay is added between two PWM signals. Since the reference clock for PWM is 10 MHz, the output frequency ($f_{0}$) from the transformer is as follows:

(3) $$f_{0}=\frac{10\;MHz}{2\times 25\;clocks}=200\;kHz$$

FPGA is also responsible for ADC sampling. The sampled ADC data are sent to the ARM module in FIFO order.

### 4.2. Device Software

**Figure 10:**
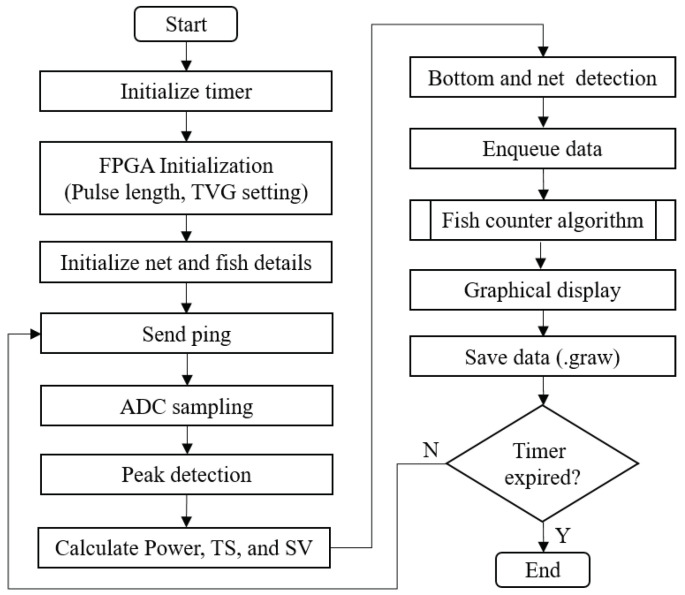
The flowchart of the device software.

The device software is the main program that controls the overall operation of SFBE. With support from other components, the software generates the ping, records ADC data, makes calculations, and displays echogram on the screen. The homebrew GUI library developed using open-source JPEG and TTF library is used for the graphical display. The flow chart of the device software is illustrated in Figure 10. The program begins by initializing a timer and hardware. A source ping is sent at a regular interval and ADC sampling of received echo is performed. From the ADC data, the received power, TS, and SV are calculated. The received power $P_{r}$ is calculated as $P_{r}=20log\left(\frac{V_{o}}{V_{i}}\right)$, where $V_{i}$ and $V_{o}$ are the input and the output voltages, respectively. Similarly, SV and TS are calculated using following equations:

(4) $$\begin{array}{cc} SV= & P_{r}+20logR-2aR-10log\left(\frac{P_{t}G_{o}^{2}\lambda^{2}}{16\pi^{2}}\right)-10log\left(\frac{c\tau \psi }{2}\right)-2S_{a\_correction} \end{array}$$

(5) $$TS=P_{r}+40logR-2aR-10log\left(\frac{P_{t}G_{o}^{2}\lambda^{2}}{16\pi^{2}}\right)$$

where $P_{t}$ is transmitter power, $G_{o}$ is transducer gain, *c* is speed of sound, λ is wavelength, τ is transmit pulse duration, ψ is equivalent two-way beam angle, and $S_{a\_correction}$ is correction factor. All data are first enqueued in a FIFO queue and later saved in a file with ”.graw" extension in a removable USB flash drive. The data are used by the fish-counting algorithm to estimate the fish count and is displayed on the screen along with the echogram. In addition, the bottom detection algorithm is implemented, which can detect and classify the sea and the net bottom based on TS and shown on the screen. The above process repeats until the timer expires, which is set to 10 min as a default value.

### 4.3. Fish Counting Algorithm

**Figure 11:**
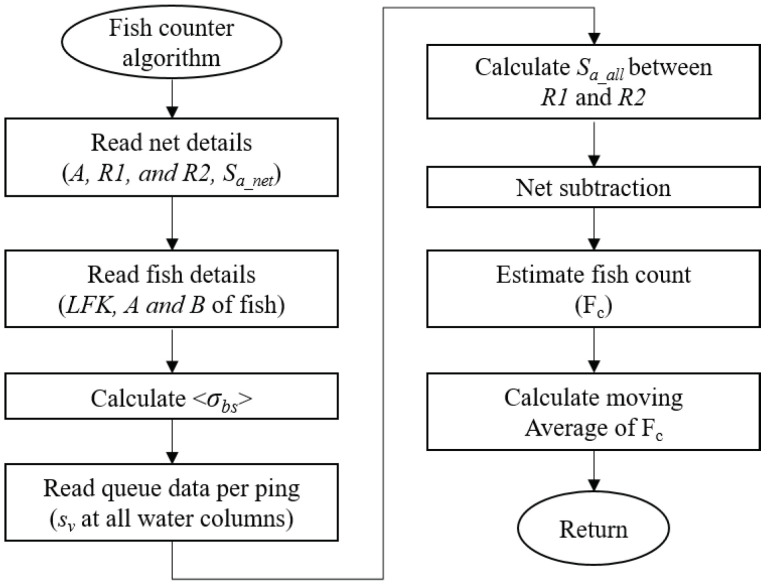
Flowchart of fish counting algorithm used in SFBE.

A novel fish counting algorithm shown in Figure 11 was developed. The basic algorithm was initially tested using a commercial echosounder and details can be found in [22]. Along with $s_{v}$ data, the algorithm takes the following input parameters. The range resolution of the echosounder denoted by dr in m, the sampling range interval measured from the top denoted by R1 m and R2 m, where R2 should be greater than the cage bottom. In addition, the fish information such as fish species, length frequency key, area of the cage, and the cage backscattering strength are required by the algorithm as inputs.

#### 4.3.1. Length Frequency Key (LFK) and Mean Acoustic Backscattering Estimation

LFK denotes sampling some fish in a cage to estimate the mean TS. LFK is a table with two columns that records the frequency of a fish with particular length, as illustrated in Table 4. The table shows the total length of fish round up to a nearest cm. The algorithm calculates $<\sigma_{bs}>$ from the provided LFK table. The TS of a fish is expressed by $TS_{L}=alog\left(L\right)+b$, where *a* and *b* are the standard coefficients unique to a fish species and *L* is fish length in cm. TS of common fish in Korea is demonstrated in Table 5 and this database is maintained by SFBE. Let *n* represent the row count of LFK table and n(L) represents the frequency of fish of length *L*; then, $<\sigma_{bs}>$ is given by

(6) $$<\sigma_{bs}>=\frac{\sum_{i=1}^{n}\left(\sigma_{Li}\times n\left(L_{i}\right)\right)}{\sum_{i=1}^{n}\left(n\left(L_{i}\right)\right)}$$

where $\sigma_{Li}=10^{\frac{TS_{Li}}{10}}$.

#### 4.3.2. Net Subtraction and Fish Counting

The signal received by SFBE is the integrated signal from fish and the net. For better accuracy, the net signal must be subtracted from the total signal. For that, a special provision is built in our system. SFBE operates in two modes: (i) empty cage mode and (ii) fish counting mode. To measure an empty net signal, SFBE is operated over the empty net on *empty cage mode* and the area back-scattering coefficient of the empty net, $S_{a\_net}$, per ping, is calculated as

(7) $$S_{a\_net}=\sum_{R1}^{R2}s_{v}\times dr$$

The moving average of $S_{a\_net}$ over pings are taken and the estimated $<S_{a\_net}>$ is saved in a configuration file. When operated in fish counting mode, SFBE first calculates $s_{a}$ between R1 and R2, denoted by $S_{a\_all}$. Then, the net subtraction is performed as follows

(8) $$S_{m}=\left\{\begin{array}{cc} s_{a\_all}-<s_{a\_net}>, & \mathrm{if}\;S_{a\_all}><S_{a\_net}>\\ 0, & \mathrm{otherwise} \end{array}\right.$$

Lastly, the fish count ($F_{c}$) per ping is calculated as

(9) $$F_{c}=\frac{S_{m}}{<\sigma_{bs}>}\times A,$$

where *A* is the area of the cage. Since $S_{m}$ divided by $<\sigma_{bs}>$ gives the fish count per m${}^{2}$, multiplying it with *A* gives the estimated fish count of the cage. The above process continues to the next ping. The average fish count is calculated by performing the moving average of $F_{c}$.

## 5. Prototyping

**Figure 12:**
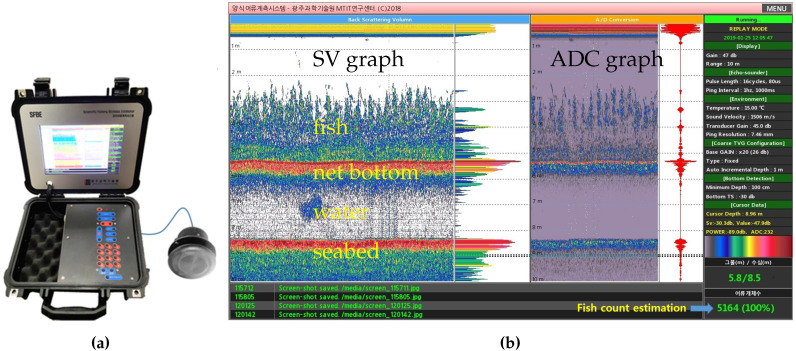
The prototype of (**a**) SFBE and (**b**) a screenshot of the echogram generated by it.

The prototype of SFBE device is shown in Figure 12a. It has a waterproof IP67 casing and weighs around 5 kg. The transducer is attached to the device via a connector. The operation of SFBE can be initiated by pressing the start button which runs the device in fish counting mode for 10 min (by default but configurable) while continuously displaying the echogram on the screen. Figure 12b shows the screenshot of SFBE displaying the echogram during an experiment. Note that the device is just a prototype and in the development phase; therefore, technical information is displayed on the right side for debugging purposes. In the figure, the left and the right half of the screen shows the SV and the ADC graphs, respectively. For both the SV and ADC graphs, the *x*-axis is time and the *y*-axis is depth or range. The pixel intensity denotes the magnitude of the signal, white representing the lowest and red representing the highest intensity. The color scale is drawn at the lower bottom part of the figure. By analyzing the pixel intensity, fish, water, net bottom, and the seabed can be differentiated in the figure. The number pair at the bottom-right corner of the screen represents the net-bottom and the seabed depths, respectively, identified by the bottom detection algorithm. The bottom-right corner displays the fish count, and (100%) is the progress bar representing the end of the operation.

### 5.1. Performance Evaluation

For the performance evaluation, two types of experiments were performed: (1) lab experiment and (2) on-site experiment. The lab experiment is performed to validate the working of SFBE by comparing it with a commercial echosounder. The on-site experiment is performed to test the fish counting performance of SFBE.

#### 5.1.1. Lab Experiment

**Figure 13:**
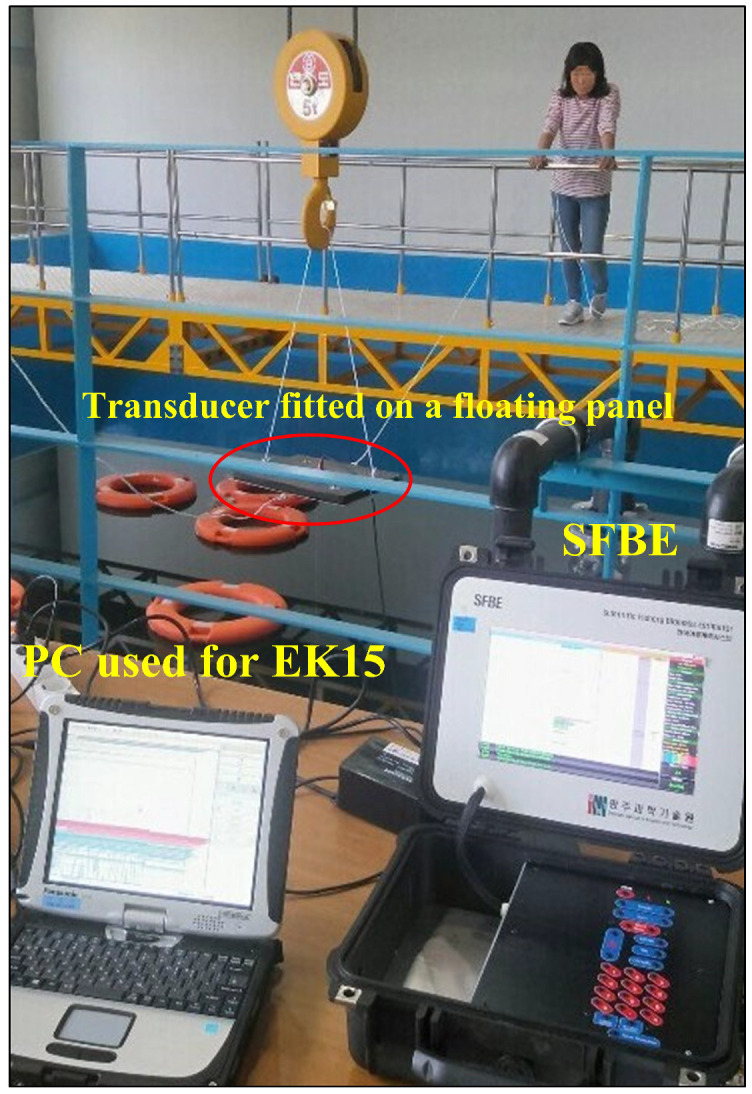
Experimental environment at KIOST. The left PC records EK15 and the right device is SFBE.

The SFBE device was compared against Simard EK15 [34]. Simard EK15 is selected because it shares similar characteristics with SFBE. Both the devices are single beam echosounder and operate in the 200 KHz frequency band. The experiments were performed at Korea Institute of Ocean Science and Technology (KIOST) in Busan, Korea [35]. KIOST is a state-run research center and has all the facilities for acoustic testing as illustrated by Figure 13. The experiments for both devices were performed under identical conditions. Both SFBE and EK15 transducers were first fitted side by side on a floating panel and the panel was placed at the center of 10 m × 10 m × 10 m tank with fresh water. At the time of the experiment, the water level was about 9.3 m. Both the devices were calibrated with the standard copper sphere of 13.7 mm and operated using the setting from Table 6. The results presented are from two sets of experiments. In the first experiment, a single target was used, whereas, in the second experiment, multiple targets were used.

**Figure 14:**
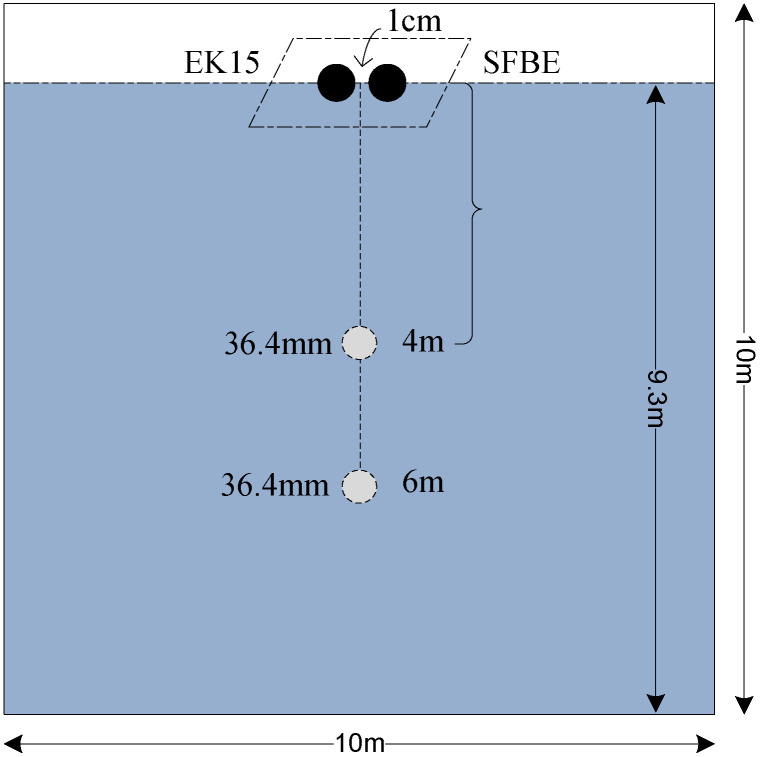
Experimental setup for the first experiment. The 36.4 mm sphere was placed at 4 m at first and then lower to 6 m.

The first set of experiments performed at KOIST is illustrated in Figure 14. A tungsten sphere of 36.4 mm was tied with a fishing line and placed at a distance of 4 m (approx. measured from the top) at first and then lower to 6 m (approx.) as shown in the figure. The experiments for EK15 and SFBE were performed separately as both the devices operate at the same frequency. The pulse duration was of 80 μs and the ping interval was of 1 s for both the devices. Repetitive experiments were carried out each lasting for 5 to 10 min. The TS data of both the devices were analyzed using Octave software [36].

**Figure 15:**
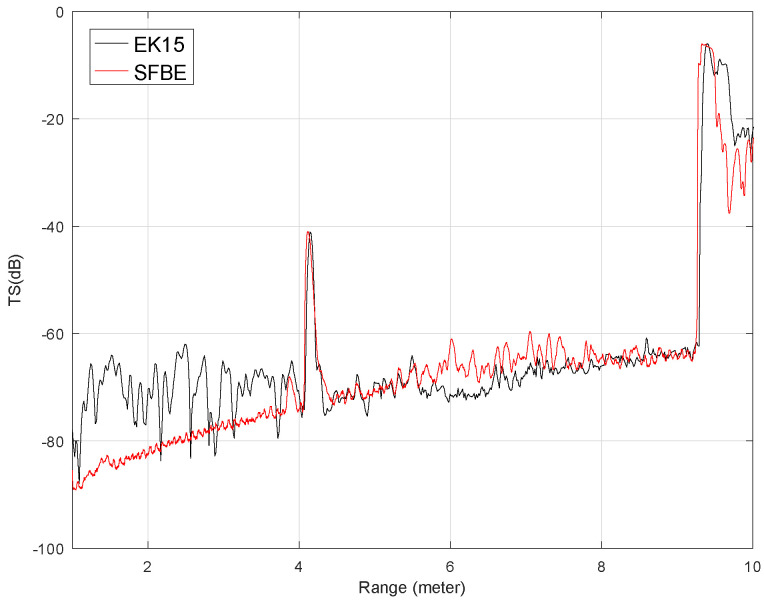
Average TS calculated for the depth range between 1 m to 10 m when the 36.4 mm sphere was at 4 m. The red graph is for SFBE and the black is for EK15.

**Figure 16:**
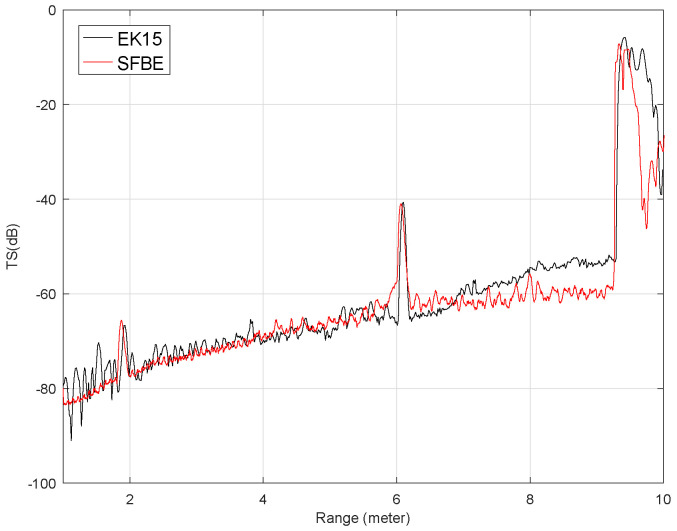
Average TS calculated for the depth range between 1 and 10 m when the 36.4 mm sphere was at 6 m.

**Figure 17:**
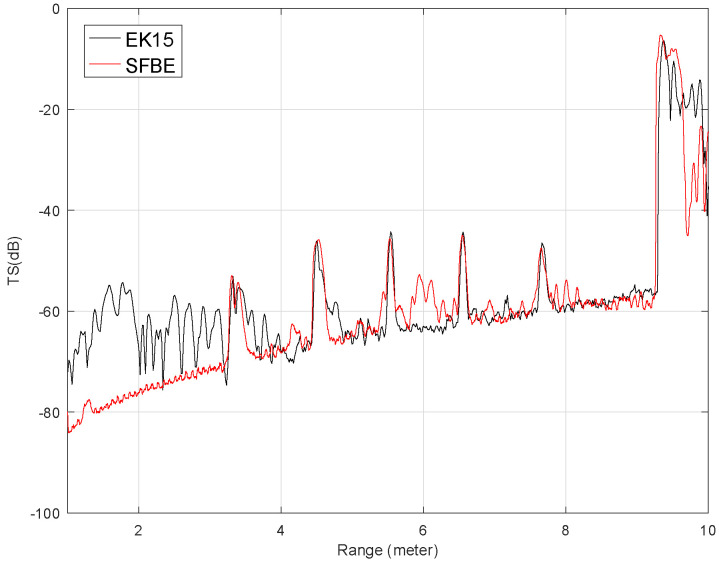
Average TS in the presence of multiple targets.

Figure 15 shows the comparison of the average TS (dB) against the range (in meter) obtained from the experiment when the sphere was at 4m. Only the signal from 1 m of depth is considered to avoid near field signals. The figure clearly illustrates that the TS is high at the target position and at the bottom but low elsewhere. Furthermore, the graph shows that the signal from SFBE matches well with that of EK15. The measured TS values from the target and from the tank bottom for both devices were of a close match. Furthermore, the target and the bottom ranges detected by both devices were also the same. The experimental results verify that the performance of SFBE is on par with EK15. However, we observed significant fluctuation in TS for EK15 at the range below 3 m which was not in the case of SFBE. Furthermore, since the range resolution of SFBE is 3.75 mm, which is much higher as compared to EK15 (14 mm), SFBE has better signal resolution and characterization of targets near boundaries [37]. The superiority of high range resolution is demonstrated by the reflected signal from the bottom of the tank in all the experiments. As can be seen in Figure 15, Figure 16 and Figure 17, the range graph of the tank bottom is perpendicular for SFBE, but is slightly inclined in the case of EK15.

Similarly, Figure 16 shows the comparison of the average TS vs. range for the sphere placed at 6 m. The results obtained were similar to the previous experiment. Furthermore, in both the experiments, the calculated average TS values of the 36.4 mm sphere were almost the same. The results from both experiments show that the noise level of SFBE is much less than that of EK15, especially at the lower depths. Since EK15 is designed to operate at higher depth, it could be the reason for the higher noise level at lower depth.

**Figure 18:**
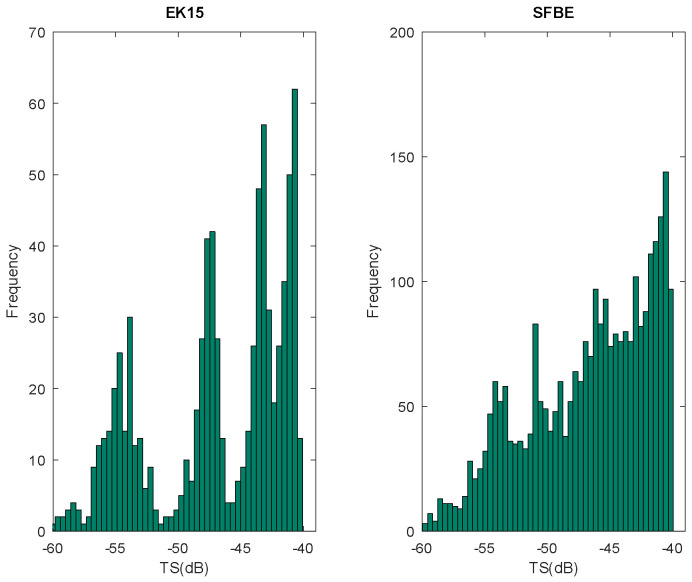
Histogram of TS measured around the target for EK15 (**left**) and SFBE (**right**).

In order to further analyze the received signal from both the devices, the signals near the target are further scrutinized. The data from the target at 4 m is used for this analysis. By analyzing the data from both the devices, we observed that the target was at 4.15 m. Therefore, the TS values between 4.1 and 4.2 m were used to plot the histogram shown in Figure 18. The left histogram is for EK15 and the right is for SFBE. As seen in the figure, the TS signal is smooth with linear growth in the case of SFBE. However, in the case of EK15, we can observe peaks and valleys. The reason for the smooth signal in the case of SFBE could be because of better range resolution, which is also illustrated by the high frequency in the histogram. The measured TS values of 36.4 mm tungsten sphere by both the devices are shown in Table 7. The reference TS of the sphere in the freshwater is –39.8 dB [30]. The result clearly demonstrates that the TS measured by SFBE is closer to the reference value.

**Figure 19:**
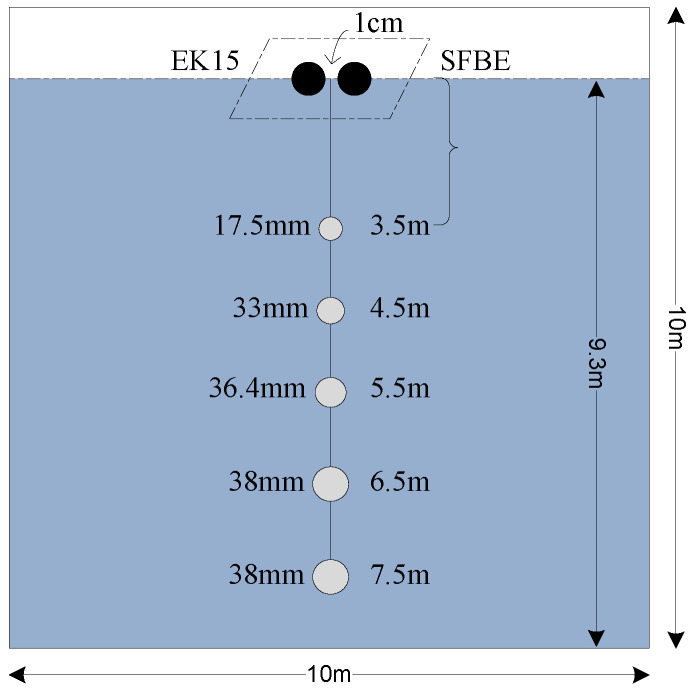
Experimental setup for the second experiment. Five spheres were tied together by a string with a separation of 1 m.

In the second experiment, five tungsten spheres (17.5 mm, 33 mm 36.4 mm, and two 38 mm) were tied together with a fishing line in the ascending order of their sizes and hanged down from the middle of the floating panels as illustrated in Figure 19. The first sphere (17.5 mm) is at the distance of 3.5 m from the top and the separation between any two adjacent spheres were of 1 m. The objective of this experiment is to compare the performance of SFBE with EK15 in the presence of multiple targets.

Figure 19 shows the comparison of the average TS (dB) against range (in meter) obtained from the second experiment. Here, also, the signal from both devices matches well. The maximum power strength at each target position of SFBE matches well with that of EK15. Similarly, the TS and the range detected for the tank bottom by both the devices were also the same. However, in this experiment, we also observed significant fluctuation of signal in EK15 at the range below 3 m, which was not the case for SFBE.

#### 5.1.2. On Site Experiment

On-site experiments were performed on a commercial offshore fish farm in the sea, which is located in Wando of South Korea. The farm consists of many cages made on a floating grid. Five cages (named Cage1, Cage2, Cage3, cage4, and cage5) were selected for the experiment and their details are shown in Table 8. The cages are made of nylon nets each having dimensions of 7 m × 7 m × 7 m with an 11 mm square mesh. In all five cages, rockfish were farmed. A total of 30 to 40 fish were sampled and recorded in their respective LFK tables. The fish abundance shown in Table 8 was obtained from the farm’s records. Our findings from the previous study [22] suggest that the accuracy of the algorithm is high during dark hours of the lowest current day at the slack time. The experiments were performed from 2020.01.19 to 2020.01.20 (low water current), for two days after sunset and during slack water [38]. The fish in all cages were more than two years old. Before the fish counting experiment, the empty net analysis was performed on an empty net cage to estimate $<S_{m\_net}>$. The empty net analysis was performed in a separate cage but had the same age and material as of the five cages. The same $<S_{m\_net}>$ was used for net subtraction in all experiments. The experiments were repeated five times in each cage. The fish count result was divided by the volume of the net (343 m${}^{3}$) to produce an estimated density (fish/m${}^{3}$).

The estimated fish densities were compared to actual densities using a linear mixed-effects model with cage as random effects using statsmodels (v0.11.1) Python module [39]. In addition, *t*-tests were performed to test the differences in the observed slope and the hypothesized slope = 1 and the observed intercept and the hypothesized intercept = 0. The significance was evaluated with α < 0.05. The relative standard error (RSE) was calculated from each set of five repeated measurements and was tested to determine if RSE was consistent across true fish densities using simple linear regression [39]. Similarly, root mean square error (RMSE) for each experiment was observed.

**Figure 20:**
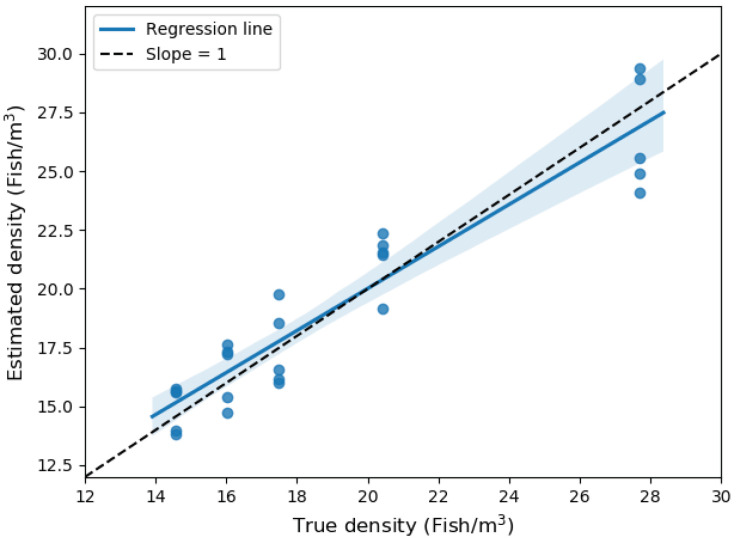
The regression line of estimated fish density (fish/m${}^{3}$) at various fish densities. The shaded area represents the 95% confidence interval of the slope and the dash line with (slope = 1) would represent 100% accuracy.

**Figure 21:**
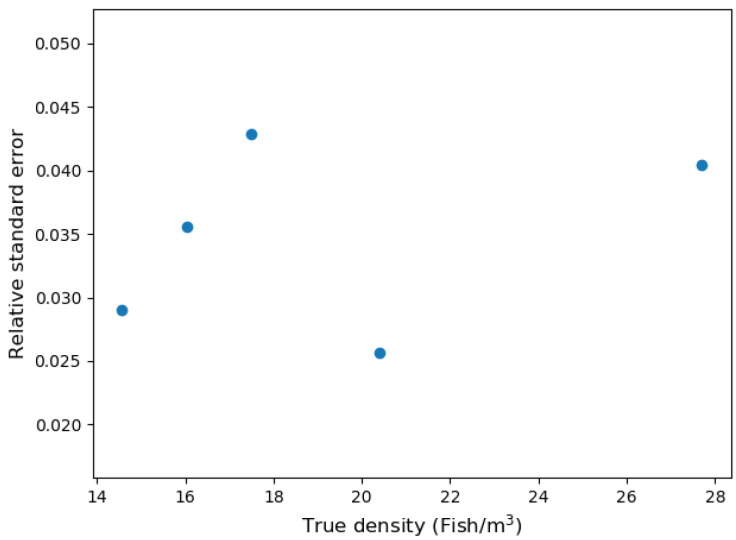
The relative standard error (SE/mean) for the density estimation at various fish densities.

Figure 20 compares the estimated and true density. As can be seen in the figure, the estimated density was directly proportional to true density (slope = 0.93 and intercept = 1.5). The observed slope was not significantly different from 1 (t = 0.18, d.f. = 1, P = 0.88), and the intercept was also not significantly different from 0 (t = 0.14, d.f. = 23, P = 0.88). In addition, RSE depicted in Figure 21 did not show any trend as density is varied (F${}_{1,3}$ = 0.32, P = 0.61). The observed average, maximum, and minimum RMSE were 7.36%, 13.52%, and 4.19%, respectively. The results suggest that SFBE device can accurately estimate fish count with an average error of ±7%. The estimated slopes were less than one (although not statistically different than one). This was likely caused by an underestimation of fish abundance at high densities. Since our algorithm does not consider the shadowing effect, it might have contributed to underestimation [39].

The experimental results presented are based on a single fish species on fixed cage size. Therefore, more tests are required to validate the performance of SFBE. In the future, more experiments with different fish species and various cage sizes will be performed.

## 6. Conclusions

A cost-effective, compact, portable but advanced echosounder with an objective of estimating fish count in a closed farming net was designed, developed, and tested. The authenticity of the data generated by our device was verified by comparing it with a popular commercial echosounder. The experimental results verify that the data generated by our device is valid and on par with the commercial device. Furthermore, the accuracy of fish count estimated was tested by performing experiments on cages with know fish counts. The on-site experimental results demonstrated that our device can accurately estimate fish count. Our device could be used for fish management on farms.

## Figures and Tables

**Table 1:** ARM Module Specification.

Parameter	Value
CPU	S5P6818, 1.4 GHz, Octa-cores
DDR3 RAM	1GB
Connectivity	Gbps Ethernet port
USB Host	USB 2.0 Type A × 1
HDMI	HDMI 1.4A, micro HDMI(Type-D), 1080P60
GPIO	2.54mm spacing 40pin, includes UART, SPI, I2C, PWM, IO etc.
RTC	RTC Battery Seat
PCB Size	75 × 40 mm
OS	Linux Kernel 4.4

**Table 2:** Pin description of ARM Module.

Pin#	Name	Pin#	Name
1	SYS_3.3V	2	VDD_5V
3	I2C0_SDA	4	VDD_5V
5	I2C0_SCL	6	DGND
7	GPIOD8/PPM	8	UART3_TXD/GPIOD21
9	DGND	10	UART3_RXD/GPIOD17
11	UART4_TX/GPIOB29	12	GPIOD1/PWM0
13	GPIOB30	14	DGND
15	GPIOB31	16	GPIOC14/PWM2
17	SYS_3.3V	18	GPIOB27
19	SPI0_MOSI/GPIOC31	20	DGND
21	SPI0_MISO/GPIOD0	22	UART4_RX/GPIOB28
23	SPI0_CLK/GPIOC29	24	SPI0_CS/GPIOC30
25	DGND	26	GPIOB26
27	I2C1_SDA	28	I2C1_SCL
29	GPIOC8	30	DGND
31	GPIOC7	32	GPIOC28
33	GPIOC13/PWM1	34	DGND
35	SPI2_MISO/GPIOC11	36	SPI2_CS/GPIOC10
37	AliveGPIO3	38	SPI2_MOSI/GPIOC12
39	DGND	40	SPI2_CLK/GPIOC9

**Table 3:** Input/output clocks to/from FPGA.

Clock	I/O	Purpose	Remarks
CLK_IN	in	Source Clock	input from 50 MHz oscillator
10 MHz	out	PWM	200 kHz acoustic wave
12 MHz	out	ADC	SPI clock for ADC Sampling
40 MHz	out	TVG	SPI clock for TVG Control
200 MHz	out	Communication	SPI clock for communication with ARM module

**Table 4:** Length frequency key of sampled fish from Cage2.

Total Length (cm)	Frequency
25	6
26	7
27	10
29	9
30	9

**Table 5:** TS at 200 kHz of some common fish farmed in Korea.

Scientific Name	Common Name	TS Value	Reference
*Sebastes ruberrimus*	RockFish	20log(L)−72.80	[31]
*Pagrus major*	Red sea bream	20log(L)−74.10	[24]
*Acanthopagrus schlegelii*	Black porgy	20log(L)−66.89	[32]
*Planiliza haematocheilus*	Redlip Mullet	20log(L)−66.33	[33]

**Table 6:** Device setting used in experiments.

Parameter	Value
Frequency	200 kHz
Pulse duration	80 μs
Ping interval	1 s
Experiment duration	5 to 10 min

**Table 7:** TS (dB) of 36.4 mm tungsten sphere in fresh water.

Frequency	Device	Reference TS	Measured TS
200 kHz	EK15	−39.8	−40.08
	SFBE		−39.93

**Table 8:** Details of fish cages used in the experiments.

Cage	Total Length (cm)	Abundance	Density(fish/m${}^{3}$)
Cage1	26 to 31	5000	14.57
Cage2	25 to 30	5500	16.03
Cage3	28 to 32	6000	17.49
Cage4	23 to 27	7000	20.40
Cage5	24 to 29	9500	27.69
